# The Quantum Nature of Drug-Receptor Interactions: Deuteration Changes Binding Affinities for Histamine Receptor Ligands

**DOI:** 10.1371/journal.pone.0154002

**Published:** 2016-05-09

**Authors:** Mojca Kržan, Robert Vianello, Aleksandra Maršavelski, Matej Repič, Maja Zakšek, Kristina Kotnik, Estera Fijan, Janez Mavri

**Affiliations:** 1 Department of Pharmacology and Experimental Toxicology, Faculty of Medicine, University of Ljubljana, Ljubljana, Slovenia; 2 Computational Organic Chemistry and Biochemistry Group, Ruđer Bošković Institute, Zagreb, Croatia; 3 Laboratory for Biocomputing and Bioinformatics, National Institute of Chemistry, Ljubljana, Slovenia; University of Parma, ITALY

## Abstract

In this article we report a combined experimental and computational study concerning the effects of deuteration on the binding of histamine and two other histaminergic agonists to ^3^H-tiotidine-labeled histamine H2 receptor in neonatal rat astrocytes. Binding affinities were measured by displacing radiolabeled tiotidine from H2 receptor binding sites present on cultured neonatal rat astrocytes. Quantum-chemical calculations were performed by employing the empirical quantization of nuclear motion within a cluster model of the receptor binding site extracted from the homology model of the entire H2 receptor. Structure of H2 receptor built by homology modelling is attached in the supporting information (S1 Table) Experiments clearly demonstrate that deuteration affects the binding by increasing the affinity for histamine and reducing it for 2-methylhistamine, while basically leaving it unchanged for 4-methylhistamine. *Ab initio* quantum-chemical calculations on the cluster system extracted from the homology H2 model along with the implicit quantization of the acidic N–H and O–H bonds demonstrate that these changes in the binding can be rationalized by the altered strength of the hydrogen bonding upon deuteration known as the Ubbelohde effect. Our computational analysis also reveals a new mechanism of histamine binding, which underlines an important role of Tyr250 residue. The present work is, to our best knowledge, the first study of nuclear quantum effects on ligand receptor binding. The ligand H/D substitution is relevant for therapy in the context of perdeuterated and thus more stable drugs that are expected to enter therapeutic practice in the near future. Moreover, presented approach may contribute towards understanding receptor activation, while a distant goal remains *in silico* discrimination between agonists and antagonists based on the receptor structure.

## Introduction

G-protein coupled receptors (GPCR) are a family of septahelix transmembrane (TM) proteins found in eukaryotic organisms, which represent one of the main targets for drug action. There are at least 800 GPCR in the human body [1]. GPCR have two main functions: ligand binding and signal propagation. In order to initiate downstream signal transduction leading to a receptor-mediated effect, a ligand-induced or a ligand-stabilized conformational change in the GPRC, which interacts with guanine nucleotide–binding proteins (G-proteins), is necessary. Most GPCR show some constitutive activity even in the absence of the ligand bound to them; ligands are described as agonists if they are capable of showing full efficacy, partial agonists show only partial biological response, antagonists if their binding to receptor does not involve any change of basal receptor activity, or inverse agonist, a ligand with negative efficacy. From the thermodynamic point of view, the binding of antagonists to their targets is usually associated with more favorable interaction free energy (affinity) with the receptor than agonists.

Evidence suggests that agonist’s binding to GPCR is a stepwise process involving one or more conformational changes in the receptor [2,3]. A bound agonist initiates miniature conformational changes in key residues (so called molecular switches) [4] leading to more pronounced conformational changes, e.g. photostimulation induced rotation and tilting of TM6 relative to TM3 of the rhodopsin receptor [5]. Similar movements of TM6 were observed after agonist-induced activation of adrenergic receptor β2 [6], muscarinic receptor M3 [7]. Recently, impressive progress in GPCR structure determination and understanding its function has been made [8–17].

The TM domains of GPCR are held together in the basal state by a network of non-covalent chemical bonds between side chains. Any compound that specifically disrupts these intermolecular arrangements after binding has receptor activity. The binding of ligands to receptors is to a large extent controlled by hydrogen bonding and involves hydrogen bonds in ligand-receptor and ligand-water interactions, as well as receptor intramolecular hydrogen bonding and intermolecular hydrogen bonds between water molecules. Replacement of (exchangeable) hydrogen atoms by deuterium alters the hydrogen bond strength and the delicate balance between the active and inactive receptor conformation is thereby distorted.

Computational methods have made a tremendous step forward in recognizing active sites and the rational design of potential drugs. In contrast to the design of enzyme inhibitors and/or ion channel blockers, discrimination between agonist and antagonist binding to GPCR by computational methods is still in its infancy. In their critical review concerning computational methods of receptor-ligand interaction applied to olfactory receptors, Don and Riniker strongly emphasized that only Quantitative Structure Activity Relationship (QSAR) methods are currently able to distinguish between agonist and antagonists [18]. Tehan et al. in their critical compilation of available structural data for GPCR showed the relevance of specific interactions and the mobility of the trans-membrane helices in the process of receptor activation [19].

In the present work we critically examined the relevance of hydrogen bonds in the ligand-histamine H2 receptor interaction. Therefore, we replaced water in the incubation medium with deuterium oxide (heavy water) and performed saturation and displacement binding experiments using histamine, 2-methylhistamine and 4-methylhistamine, which are all histamine H2 receptor agonists. In this way exchangeable N–H and O–H protons were exchanged by deuterium, while C–H hydrogen atoms were not.

Deuteration-induced changes in the length and strength of the hydrogen bonds did not cause any statistically significant difference in the maximal binding capacities (Bmax) and equilibrium dissociation constant (*K*_D_) of 3H-tiotidine (antagonist). In contrast, the affinities of all agonists, 2-methylhistamine and histamine in particular, towards ^3^H-tiotidine labeled histamine H2 receptors binding sites were changed, confirming the relevance of hydrogen bonding in the process of agonist-receptor binding. Altered hydrogen bonding strengths were rationalized in terms of the Ubbelohde effect [20]. The computational study involved the construction of a homology model of the H2 receptor (the structure is deposited in the supporting information, S1 Table) and quantum chemical modeling of the binding free energies with included simplified quantization of the proton motion in order to assess the influence of hydrogen isotopes on the binding of histamine, an endogenous agonist. The corresponding scheme was also applied for the calculation of the histamine hydration energy that involved specific water molecules.

## Materials and Methods

### Materials

All culture material, except fetal bovine serum (Lonza, Belgium), was purchased from Gibco, USA. ^3^H-tiotidine (specific activity 82.2 Ci/mmol) was purchased from New England Nuclear, USA, D_2_O from Cambridge Isotope Laboratories, USA, histamine from Sigma, and cimetidine from GSK, Great Britain.

### Astrocyte cultures

All test animals were used in accordance with the National Institutes of Health Guidelines for the Care and Use of Laboratory Animals and Permission for Use of Laboratory Animals 34401-1/2010/8 and U34401-12/2013/3 issued by the Administration of the Republic of Slovenia for Food Safety, Veterinary and Plant Protection to prepare primary astrocytes cultures from newborn not-treated rat. 3 day old rats were sacrificed by decapitation, followed by removing the brain cortices. Primary cultures of astrocytes were prepared and cultured as previously described [21]. Cells were grown in high-glucose Dulbecco's modified Eagle's medium (DMEM), containing 10% fetal bovine serum (FBS), 1 mM pyruvate, 2 mM glutamine, and 25 μg/ml gentamycin in 95% air-5% CO_2_. Confluent cultures were shaken at 225 rpm overnight and the medium was changed the next morning; this was repeated a total of three times. After the third overnight shaking, the cells were trypsinized and cultured for 24 h in 10 μM cytosine arabinoside. After reaching confluence again the cells were subcultured into 12-well clusters and grown until becoming confluent again. The confluent cells were detached, washed twice in PBS, and cell sediment was stored at –70°C until used for the binding assay.

### ^3^H-tiotidine saturation binding assay

0.1 mg of astrocyte cell protein was incubated for 15 minutes at 25°C in 0.15M Na^+^/K^+^ -phosphate buffer (pH 7.4) or in 0.15M Na^+^/K^+^ -phosphate buffer made using D_2_O instead of miliQ water (pH 7.4) in a total volume of 0.25 mL with the indicated concentrations of ^3^H-tiotidine (1–15 nM), with a specific activity of 82.2 Ci/mmol. Specific binding represented the difference between total and non-specific binding (binding in the presence of 1 μM cimetidine).

### Competition binding assay

In the competition binding experiments, astrocyte cells were incubated together in the presence of different histaminergic ligands in the concentration range from 0.01 nM– 100 μM under the same incubation conditions as above. The concentration of ^3^H-tiotidine used was 5 nM. The binding reaction was rapidly terminated and the bound ligand was separated from the unbound by vacuum filtration. The radioactivity trapped on the GF/C glass fiber filters was counted in a liquid scintillation counter (Microbeta, Perkin Elmer, USA).

### Data analysis

The binding experiments were routinely carried out in triplicate and each experiment was repeated at least twice. The results are expressed as means ± SEM. The binding parameters (*K*_D_, B_max_, and IC_50_) were calculated by a non-linear regression method using software Prism6 version 6.00, GraphPad Software Inc. (San Diego, USA). A comparison of the data among the groups was carried out using Student`s unpaired t-test with Welch correction, where two groups of data were compared. Differences were considered significant at p < 0.05.

## Results

### Binding studies

**Fig 1:**
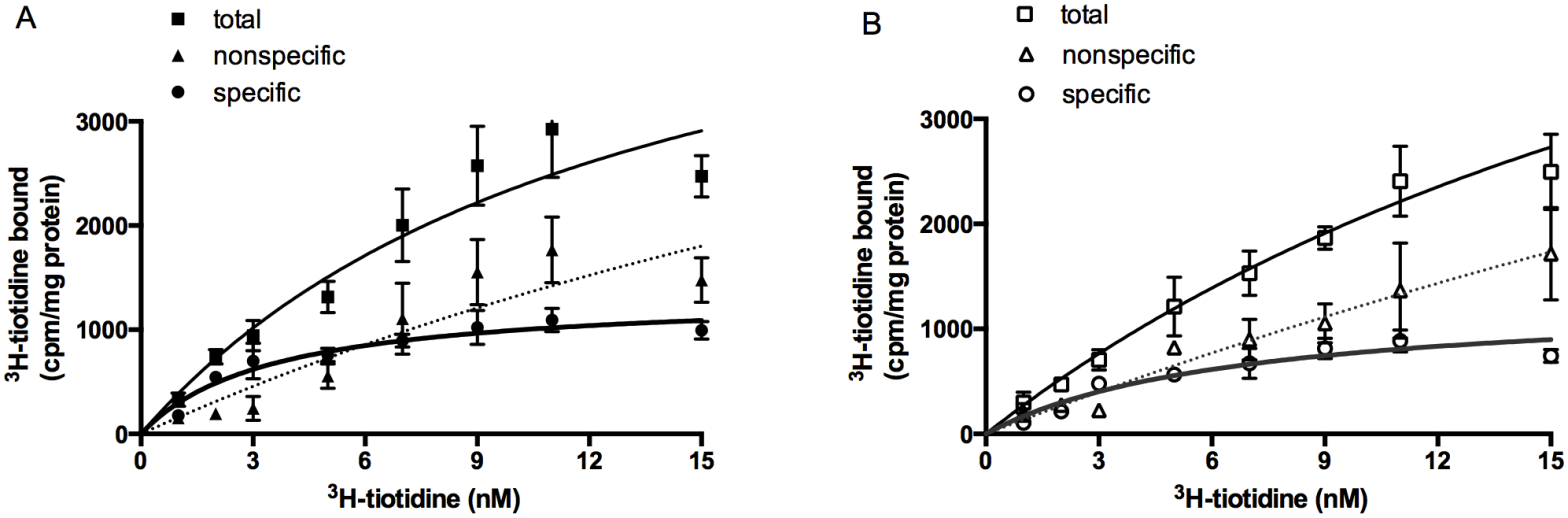
Binding of ^3^H-tiotidine to cultured neonatal rat astrocytes. Astrocytes were incubated for 15 minutes with the indicated concentrations of ^3^H-tiotidine in regular phosphate buffer (A) or in phosphate buffer made in D_2_O (B). Results are presented as a mean value ± SEM of two experiments carried out in triplicate (n = 6).

We used radio-ligand binding studies and ^3^H-tiotidine as a marker to identify histamine H2 receptor binding sites present on newborn rat cortical astrocytes in culture. ^3^H-tiotidine binding to astrocyte cells was reversible, saturable and of high affinity (Fig 1).

**Fig 2:**
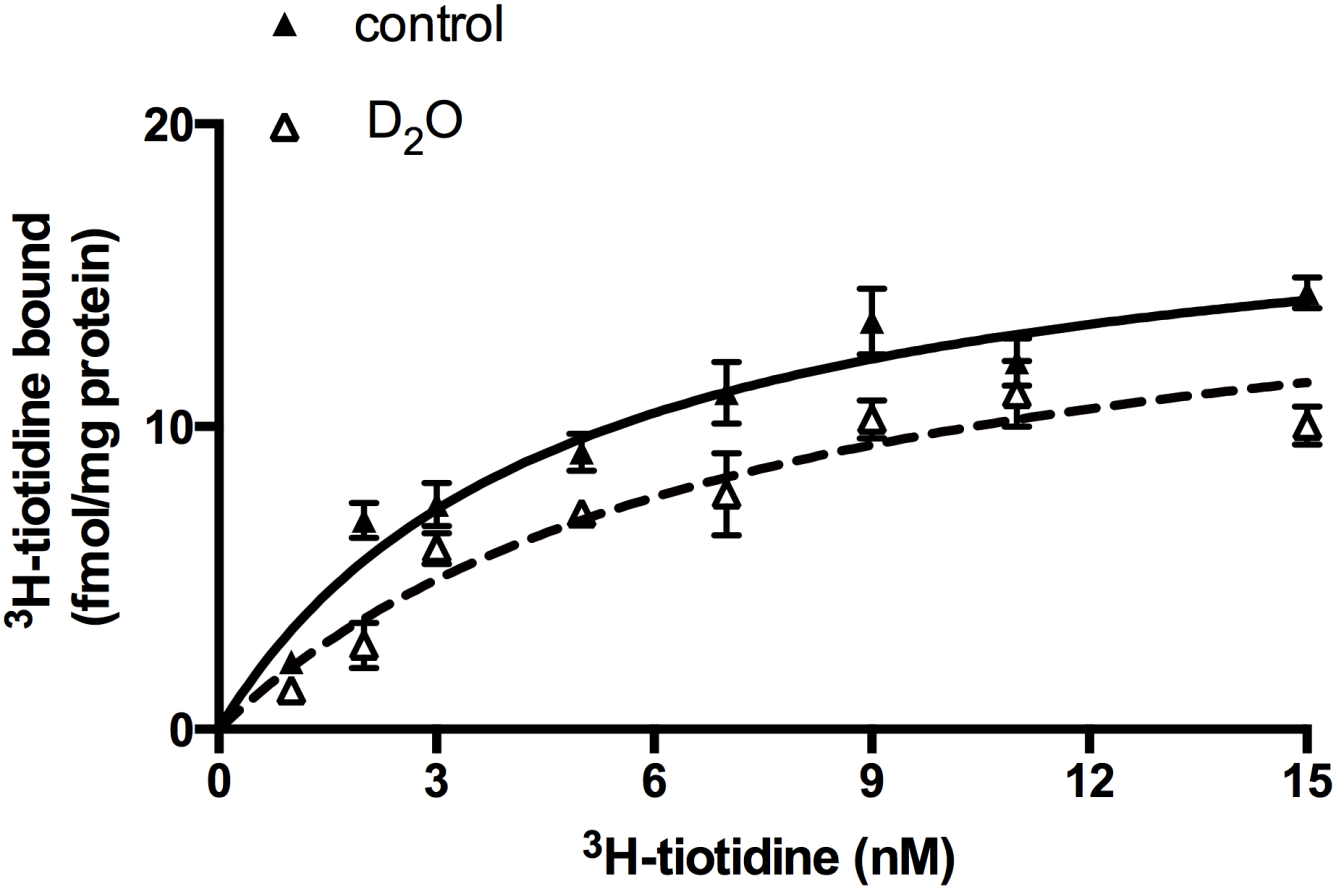
Specific ^3^H-tiotidine binding to cultured neonatal rat astrocytes. ^3^H-tiotidine binds to a single population of binding sites with a K_D_ of = 4.7 ± 1.0 nM and B_max_ of 18.6 ± 1.7 fmol/mg protein. The deuterated medium does not significantly change the binding parameters of ^3^H-tiotidine to astrocytic H2 receptor binding sites—K_D_ changed to 7.4 ± 2.4 nM and B_max_ dropped to 17.1 ± 2.7 fmol/mg protein.

Non-linear regression of specific ^3^H-tiotidine binding revealed a single population of binding sites with the equilibrium dissociation constant (*K*_D_) 4.7 ± 1.0 nM and a maximal binding capacity (B_max_) of 18.6 ± 1.7 fmol/mg protein (Fig 2). The replacement of miliQ water with deuterium oxide in the incubation medium did not affect the binding characteristics of ^3^H-tiotidine to astrocyte H2 receptor binding sites. The shapes of the total and specific binding curve remained unchanged, whereas the non-specific binding became linear. The binding parameters of specific ^3^H-tiotidine binding to astrocytic H2 receptor binding sites in deuterated medium were not significantly different from the control ones, but were significantly more scattered with *K*_D_ of 7.4 ± 2.4 nM and a B_max_ of 17.1 ± 2.7 fmol/mg protein (Fig 2).

**Fig 3:**
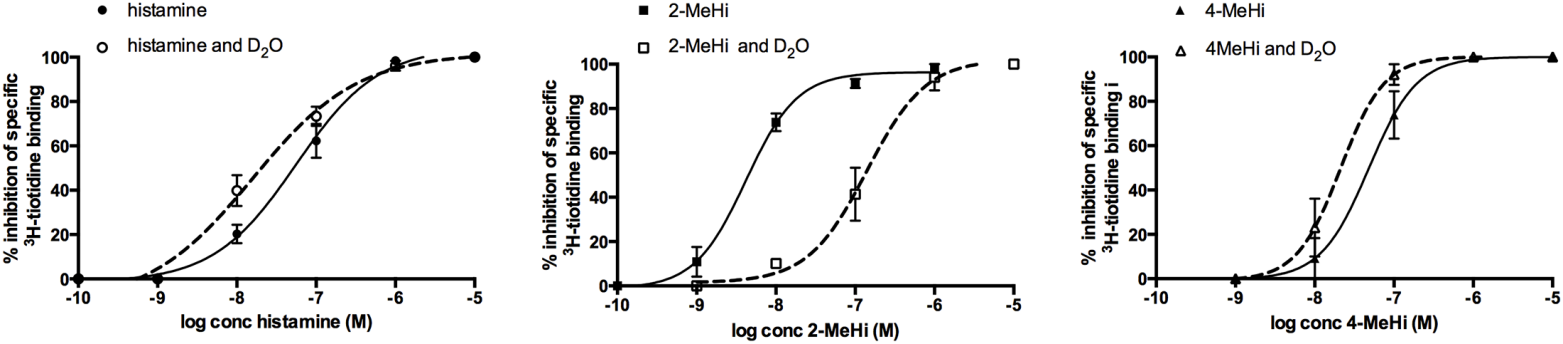
Inhibition of specific binding of ^3^H- tiotidine in cultured astrocytes with histamine, 2- and 4-methylhistamine; 2-MeHi and 4-MeHi, respectively. Deuteration significantly (p < 0.0001) decreased the pIC_50_ of 2-methylhistamine (8.38 ± 0.13 (control) to 6.85 ± 0.16 (D_2_O)), whereas it significantly (p < 0.05) increased the pIC_50_ of histamine (7.25 ± 0.11 (control) to 7.80 ± 0.16 (D_2_O)) and marginally increased the pIC_50_ value of 4-methylhistamine from 7.31 ± 0.28 (control) to 7.67 ± 0.13 (D_2_O).

In the next step, inhibition binding studies were performed using histaminergic agonists histamine, 2-methylhistamine and 4-methylhistame as displacers of specific ^3^H-tiotidine binding in both the control and deuterated environment. As shown in Fig 3, histamine and its methyl derivatives displaced the bound ^3^H-tiotidine in a concentration-dependent manner in the control and deuterated medium. The displacement curves were monophasic, with the Hill coefficients (0.9–1.4), and were best evaluated using a one-site competition curve fit. Micromolar concentrations of histaminergic agonists fully displaced the bound ^3^H-tiotidine either in the control or in the deuterated buffer.

These results clearly indicate that deuteration modulates the binding patterns by increasing the binding affinity for histamine, while reducing it for 2-methylhistamine. On the other hand, the binding of 4-methylhistamine did not change upon deuteration, at least not in the same way as for the other two compounds. This intriguing phenomenon will be rationalized by computational analysis using modern quantum-chemical calculations on a model system.

### Homology model of the histamine H2 receptor

**Fig 4:**
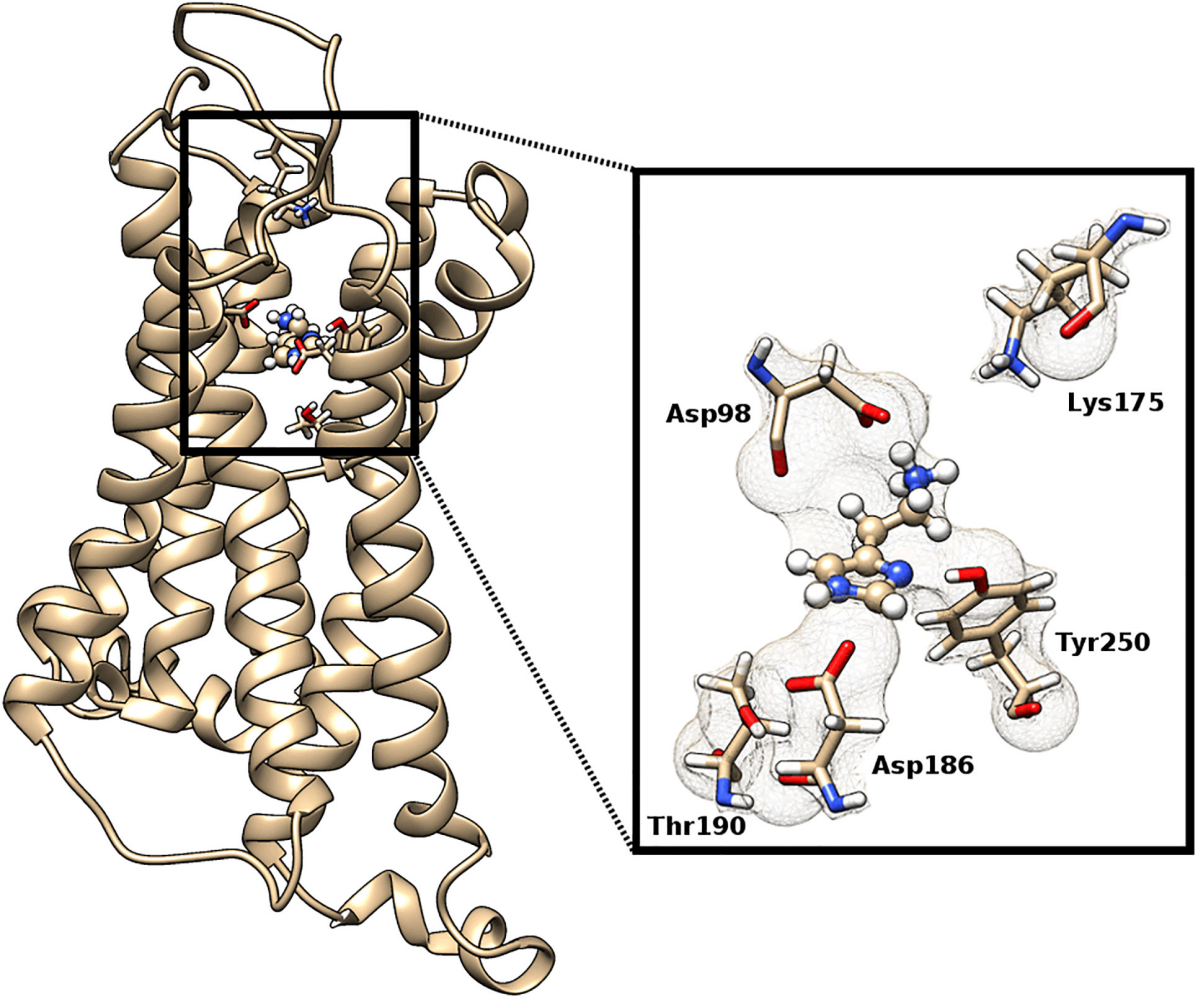
The structure of the H2 homology model with bound histamine. The incept shows the structure of the receptor model employed in quantum-chemical analysis, where the selected amino acid residues were truncated at the corresponding α–carbon atoms which positions were kept frozen during geometry optimization.

Two earlier homology models for human H2 histamine receptor (hH2HR) were developed taking into account only the crystal structure of turkey β1–adrenoreceptor [22], and using bovine rhodopsin, human β2–adrenoreceptor and human adenosine A2a receptor [23]. These receptor models were followed by the most recent structure produced using the X-ray crystal structures of turkey β1-adrenoreceptor, human histamine H1 receptor, human β2-adrenergic receptor and human D3 dopamine receptor [24]. Here, we report hH2HR model structure based on the structures of the human histamine H1 receptor (3RZE), the neurokinin 1-receptor (2KS9), human β2-adrenergic receptor (2RH1), human β1-adrenoceptor (4BVN), and M3 muscarinic acetylcholine receptor (4DAJ). The protein sequence of the hH2HR (ID P25021) was retrieved from the Universal Protein Resource (http://www.uniprot.org/) database, while its 3D structure was constructed by homology modeling tools (I-TASSER [25], MODELLER [26], SWISS MODEL [27] and Phyre2 [28]). The best model was selected using the following criteria: the statistics of non-bonded interactions between different atom types using ERRAT tool [29] and stereochemical properties using PROCHECK tool [30]. The geometry of histamine was optimized using the HF/6–31G(d) model with Gaussian 09 [31], after which the atomic partial charges were obtained by fitting the electrostatic potentials using the RESP fitting technique, and the structure manually placed in the receptor active site. Protonation states of amino acid residues were estimated by PROPKA 3.1 [32] and by visual inspection of their hydrogen-bonding patterns. Prepared model was employed as the initial structure for the molecular dynamics (MD) simulations in AMBER12 [33] using general AMBER force fields GAFF and ff14SB for histamine and receptor, respectively. The whole system was solvated in a truncated octahedral box of TIP3P water molecules spanning a 10 Å thick buffer. Protein geometry optimization was conducted in four cycles with different constraints. Optimized systems were gradually heated from 0 to 300 K and equilibrated during 50 ps, and subjected to productive and unconstrained MD simulations of 100 ns in length at constant pressure (1 atm) and temperature (300 K), the latter employing the Langevin thermostat with a collision frequency of 1 ps^–1^. Bonds involving hydrogen atoms were constrained using the SHAKE algorithm [34]. The Particle Mesh Ewald method was applied to calculate long–range electrostatic interactions. The nonbonded interactions were truncated at 10.0 Å. The final structure is shown in Fig 4 together with the truncated model used in the subsequent quantum-chemical analysis.

### Quantum-chemical calculations and the quantization of the nuclear motion

After building a homology model of the histamine H2 receptor and performing molecular dynamics simulations with bound histamine, we identified three residues crucial for histamine binding as Asp98, Asp186 and Tyr250, which bind histamine to its ethylamino group, N–H moiety on the imidazole ring and imino nitrogen atom of the same group, respectively (Fig 4). It is very important to emphasize that this differs from the model by Birdsall and co-workers [35] proposed on the basis of the site-directed mutagenesis studies carried out by Gantz et al. [36], which suggested Thr190 to bind histamine on its imino nitrogen. However, Thr190 is not in a position for such interaction since it is found in the vicinity of the Asp186 residue and hydrogen bonded to it (Fig 4), while Tyr250 is ideally positioned to form O–H·····N bonding. To make sure that we have the right histamine orientation within the receptor, we repeated MD simulations starting from five different histamine orientations including the one where the imidazole ring is rotated by 180 degrees around the N(ring)–C(β) bond. All five MD simulations produced the same structure as depicted in Fig 4 as the most dominant. In order to enrich our model, we also included Lys175 residue which is found in the vicinity of Asp98 with the possibility to form hydrogen bonding interactions.

Protonation states of the enzyme and receptor binding sites are always associated with uncertainty. This is an important issue since the binding affinities depend heavily on the corresponding protonation states. PROPKA analysis performed on the homology structure yielded the following values: p*K*_a_(Asp98) = 6.0, p*K*_a_(Lys175) = 10.9, and p*K*_a_(Asp186) = 5.7. Accordingly, the protonation states of Asp residues were assigned to both have a negative charge, while Lys175 and histamine were considered as monocations, the latter in the most stable π–tautomeric form. This setup allowed for the formation of a salt bridge between the histamine amino group and the Asp98 residue. The initial geometries of the considered residues were extracted from the lowest-energy MD snapshot and truncated at the corresponding α–carbon atoms. The positions of these α–carbons were then kept frozen during the subsequent quantum-mechanical analysis.

We optimized the constructed complex with the DFT M06-2X model in conjunction with the 6–31+G(d,p) basis set, and total molecular electronic energies were extracted without thermal corrections, thus, the results reported in this work correspond to differences in the electronic energies. The selection of this model was prompted by its success in providing accurate thermodynamic and kinetic parameters for organic systems, being particularly efficient in treating nonbonding interactions [37–39]. Solvation effects were taken into account by employing the CPCM polarized continuum model of Tomasi and coworkers [40]. Since the dielectric constant of the internal protein environment is a subject of heated debates [41], we employed the value of ε = 4.0 as suggested by Himo and coworkers [42]. To gauge the obtained set of results we repeated the analysis using the B3LYP functional and a dielectric constant of ε = 20.0; therefore, four set of values are reported here. A dielectric constant of ε = 78.36 was employed to model the corresponding binding affinities in aqueous solution.

To accurately calculate the binding affinity of histamine to the receptor one must bear in mind that histamine starts in the aqueous medium and is then moved to the internal receptor binding site, which is surrounded by the rest of the protein. Therefore, the thermodynamics involve two components: the energy of hydration and the energy of interaction with the receptor binding site. Thus, the binding affinities were calculated from the following equation:

$$E_{binding}=E_{complex}^{\varepsilon =protein}-\left(E_{histamine}^{\varepsilon =water}+E_{receptor}^{\varepsilon =protein}\right)$$

Quantum-chemical calculations employing the classical treatment of nuclear motion have an inherent flaw when it comes to predicting the geometries of deuterated species. Since the mass of the nuclei has no impact on the geometry optimization, the geometry predicted for deuterium and hydrogen is identical, which is physically not entirely correct. Methods for the quantization of nuclear motion have been developed; nevertheless, they are limited to only a few degrees of freedom. In our case we have several critical protons directly involved in the hydration process and in the recognition of histamine by the H2 receptor. Therefore, we decided to proceed with an approximate treatment of the nuclear quantum effects involved in binding. We expect that the majority of the H/D nuclear quantum effect originates from the shortening of the donor X–D bonds relative to the X–H (X = N, O) bonds, giving rise to elongated donor-acceptor distances among heavy atoms upon deuteration, which is known as the Ubbelohde effect [20]. In other words, the attenuation of hydrogen bonding by deuteration can be rationalized in terms of the dipole-charge interaction. Since the donor-proton bond is longer for H than for D, it possesses a higher dipole moment giving rise to more favorable interaction with the negative charge on the proton acceptor. Recently, Bordallo and coworkers performed a very accurate neutron diffraction study of alanine zwitterion and they demonstrated that N–D distances shrink by 2.3% relative to the N–H distances [43]. We used this constraint in the present study to approximately model the effects of deuteration on binding. Specifically, we performed a full relaxed geometry optimization for systems corresponding to H, and then scaled all the acidic N–H and O–H distances down by 2.3% and kept them frozen during the geometry optimization for deuterated species. It was essential to perform the same scaling in the calculation of histamine in aqueous solution as well.

### Calculations support changed affinity upon deuteration

**Fig 5:**
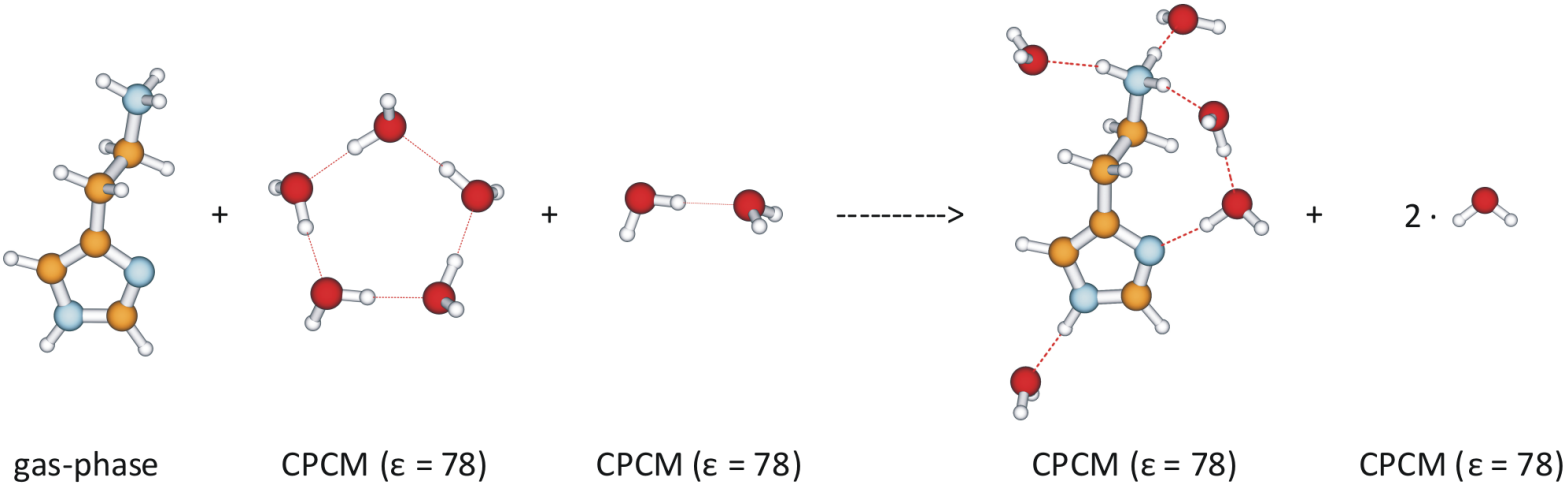
Computational scheme of histamine monocation interacting with five water molecules to calculate the free energy of hydration. The choice of the dielectric constant in the computations is indicated in round brackets.

In the previous set of results [44], we established that the most convenient way to calculate the free energy of solvation, Δ*G*_HYDR_, of histamine monocation is to use the scheme shown in Fig 5, since we demonstrated that it requires at least five explicit water molecules to correctly reproduce the stability of histamine conformers and tautomers in aqueous solution.

The scheme considers five water molecules as a pentamer in the aqueous solution, which conserves the number of hydrogen bonds on both sides of the equation, being in a full analogy with the concept of homodesmotic reactions [45]. It gives the hydration free energy of –67.5 kcal mol^–1^ in H_2_O, being in a very close quantitative agreement with the MP2/6–31++G(2d,2p) and Langevin dipole calculated value of –68.6 kcal mol^–1^ [46], which lends credence to the computational model applied here.

**Fig 6:**
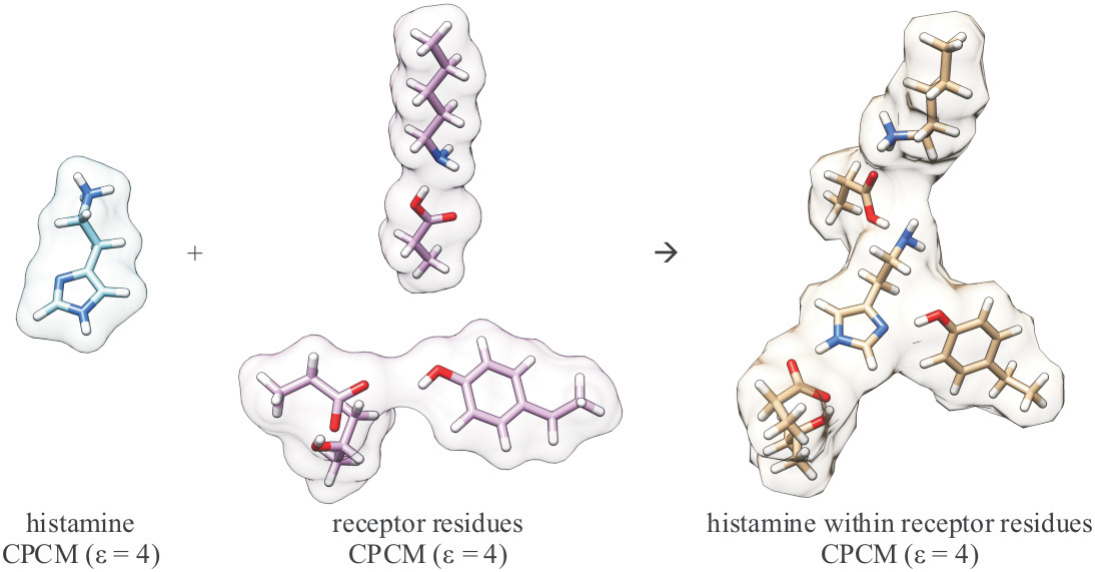
Computational scheme to calculate the interaction energy between the histamine monocation and the receptor site. All the structures were obtained on the M06–2X/6–31+G(d,p) level with CPCM solvent reaction field. Please note that beside dielectric constant of 4, a value of 20 was also considered.

Interaction energy between the histamine monocation and the receptor site was calculated using the scheme shown in Fig 6 and the results are presented in Table 1. We can define the overall change in the binding energy, ΔΔ*E*_BIND_, of histamine to the histamine H2 receptor binding site upon deuteration as a difference in the corresponding interaction and hydration energies:

$$\Delta \Delta E_{BIND}\left(H\to D\right)=\Delta E_{INTER}\left(H\to D\right)-\Delta E_{HYDR}\left(H\to D\right)$$

**Table 1:** Changes in the energy of histamine hydration (ΔE_HYDR_), H2 receptor interaction (ΔE_INTER_) and total receptor binding (ΔE_BIND_) upon deuteration.

	**dielectric constant ε = 4.0**					
	**M06–2X results**			**B3LYP results**		
	ΔE(H_2_O)	ΔE(D_2_O)	ΔΔE(H_2_O–D_2_O)	ΔE(H_2_O)	ΔE(D_2_O)	ΔΔE(H_2_O–D_2_O)
**ΔE**_**HYDR**_	–67.45	–67.39	–0.06	–64.30	–64.22	–0.08
**ΔE**_**INTER**_	–39.64	–40.09	0.45	–42.77	–42.59	–0.18
**ΔE**_**INTER**_**(calc)**	27.81	27.30	0.51	21.53	21.63	–0.10
	dielectric constant ε = 20.0					
	M06–2X results			B3LYP results		
	ΔE(H_2_O)	ΔE(D_2_O)	ΔΔE(H_2_O–D_2_O)	ΔE(H_2_O)	ΔE(D_2_O)	ΔΔE(H_2_O–D_2_O)
**ΔE**_**HYDR**_	–67.45	–67.39	–0.06	–64.30	–64.22	–0.08
**ΔE**_**INTER**_	–31.98	–32.30	0.32	–25.87	–26.17	0.30
**ΔE**_**INTER**_**(calc)**	35.47	35.09	0.38	38.43	38.05	0.38
**ΔE**_**INTER**_**(exp)**			**0.73**			

All values are in kcal mol^–1^.

It turns out that, in our model, the hydration energy for histamine is more favorable in water by 0.06 kcal/mol than in D_2_O. However, this is completely outperformed by a difference in the interaction energy with the receptor binding site, which assumes 0.45 kcal/mol in favor of the deuterated systems. In total, our model predicts that the overall binding of histamine and its deuterated analogue is stronger by 0.51 kcal/mol for the latter. This was found to be completely in line with the experimental value of 0.73 kcal/mol, which was deduced from the reported measured values of 56 nM and 16 nM for histamine and deuterated histamine, corresponding to the binding free energies of –9.86 and –10.59 kcal/mol, respectively. The difference in experimental free energies of binding upon deuteration Δ*G*_BIND_(H→D_)_ was calculated by using formula Δ*G*_BIND_(H→D_)_ *= −RT ln(K*_*D*_*/K*_*H*_*)*, where *K*_*D*_ and *K*_*H*_ are experimental ligand binding constants for species with D and H, respectively. The agreement between theory and experiment is excellent given the simplicity of the model used here for the quantization of nuclear motion performed on only a small but carefully selected part of the receptor molecule. We note in passing that our model predicts that in the receptor binding site there is a proton transfer from the charged histamine ethylamino group to the Asp98 residues producing neutral amino and carboxylic groups. This allows Lys175 to undergo large conformational change and approach Asp98 to a N(Lys175)–O(Asp98) distance of only 3.241 Å in our cluster model (Fig 6), while during MD simulations with monocationic histamine in the binding site this distance oscillates mostly between 8–13 Å. At this point we can only indicate that perhaps this large Lys175 conformational change could be involved in the receptor activation process and is certainly worth further investigations in the future.

Increasing the dielectric constant to ε = 20.0, lowers the accuracy of the M06–2X results. Interaction energies are reduced compared to values obtained with ε = 4.0, so is the overall ΔΔ*E*_BIND_(H→D) value which, at this model, assumes 0.38 kcal/mol. This strongly suggests that the interior of the H2 receptor site is probably significantly hydrophobic, which is why better agreement is reached with lower ε value. On the other hand, B3LYP functional underperforms relative to M06–2X data and is not recommended for this kind of studies. For example, with ε = 4.0, B3LYP predicts even opposite trend in binding upon deuteration in a way that the system with H binds strongly by 0.10 kcal/mol.

At the first glance, this trend in binding affinities looks counterintuitive, since deuteration reduces the strength of hydrogen bonding. The opposite changes in affinity upon deuteration for histamine can be rationalized by the different magnitude this effect exerts on all hydrogen bonds involved in the system. As a result of this complicated and interconnected balance, the affinity of histamine for the H2 receptor increases upon deuteration by 0.73 kcal/mol.

### Proposed model might be used for olfactory receptors ligand deuteration

An interesting proposal concerning the relevance of the molecular vibration-sensing component in Drosophila olfactory receptors has been reported [47]. The authors studied the olfaction of progressively deuterated acetophenon. Animals distinguished between the deuterated and nondeuterated varieties. Anosmic animals, however, did not distinguish between the isotopomers, which gave strong evidence about the interaction of the odorants with the olfactory receptors. The authors interpreted the results in terms of the spectral sense of olfaction, an approach that has been criticized by Hettinger [48] and recently by Block and co-workers [49]. We suggest an alternative interpretation of the experimental data since we believe that receptors cannot distinguish between different vibrational frequencies and associated dynamical effects. By dynamical effects we mean deviation from the transition state theory when the receptor proceeds from the inactive to active form. Receptors are proteins and, as in enzymes, dynamical effects are most probably irrelevant, [50,51] although some authors advocate the relevance of dynamical effects in enzyme catalysis [52]. We propose the following interpretation of altered olfactory receptor response upon deuteration. Follow-up of deuteration of the aromatic moiety shrinks the effective C–D distance relative to its C–H value. The aromatic C–H bonds act as proton donors and form weak hydrogen bonds with water molecules and proton acceptors at the receptor binding site. The same is true for the quadrupole moment of the aromatic moiety, which is decreased, as well dipole-quadrupole and possible cation-π interactions, which are attenuated by deuteration. As in the case of histamine, the interaction free energy with the environment is attenuated upon deuteration. We expect much smaller effects in terms of binding for apolar C–H moieties such as in acetophenon than polar groups as present in histamine. Olfaction is a physiological process involving several receptors [53,54]. Drosophila involves 62 olfactory receptor subtypes, while for humans there are estimated 900 olfactory receptors subtypes. Olfaction can be interpreted in a way that one particular odorant acts as a (partial) agonist at some olfactory receptor subtypes and/or antagonist at the other olfactory receptor subtypes. The response of a group of olfactory receptors is formally vector information that is memorized. Deuteration slightly changes the response of the entire receptor array, which is sufficient to discriminate between the isotopomers. At this point one can speculate that olfactory system would not be able to discriminate between the isotopomers with exchangeable protons because the D is rapidly exchanged by H before the odorant reaches the receptor binding site.

## Discussion

It should be clear that G–protein coupled receptor activation is a complex process, associated with large conformational changes between receptor states that are difficult to study experimentally, while at the same time too slow for direct molecular simulations. For membrane receptors, high-resolution structures of the active and inactive conformation have only recently become available and understanding the nature of GPCR receptor activation on the atomic and electronic level represents a major challenge, [19] while for nuclear receptors some progress in this direction has been made [55,56]. Even knowing the crystal structure of the H2 receptor it is very difficult to simulate the process of receptor activation by brute force molecular simulation. Recent study of β_2_-adrenergic receptor activation by Kobilka’s and Shaw’s groups gave atomic insight into this complex event [57]. It remains a question as to what is the initial step associated with ligand binding that does or does not cause receptor activation. It may well be that the initial activation event is a proton transfer at the binding site.

The development of the H2 antagonists cimetidine and ranitidine, and their analogs through various substitutions at the guanidine moiety, has shown that the proper p*K*_a_ value of the corresponding guanidine group is essential for antagonistic activity. Since p*K*_a_ values are closely related to the ability to transfer a proton, one could speculate that proton transfer associated with ligand-receptor binding can be an essential element of the H2 receptor activation. Computational methodologies [58] along with spectroscopic techniques [59,60] for studies of proton affinities and dynamics, including quantization of nuclear motion, are developed and ready to be used as soon as the crystal structure of the H2 receptor will become available. Path integration seems to be the method of choice since it allows for quantization of several degrees of freedom [61].

It remains a major challenge to perform a functional study of the deuteration effect on the histamine H2 receptor. There is an important difference between the G-protein coupled receptor binding site and a unit of the effector site of the receptor molecule. The ligand binding site communicates with the extracellular space and in this extracellular part of the receptor, protein exchangeable protons can be replaced by deuterons upon incubation in D_2_O, whereas the hydrogen atoms present in the cytosolic part of the receptor molecule cannot be exchanged with deuterons. G-protein activation is therefore not affected by deuteration. Nevertheless, during the experiment, D_2_O molecules can freely diffuse or are taken up by transporters into the astrocyte cell and G-protein activation can be affected, too. In this work we used the histamine H2 receptor as a model because its primary structure is known. We revealed the importance of hydrogen bonding in the binding of its agonists on newborn rat cortical astrocytes through experiments involving deuteration. Replacing exchangeable hydrogen with deuterium atoms leads to changes in the corresponding intermolecular and intramolecular distances of the partners involved in the hydrogen bonding, which diminishes the strength of these interactions among interacting agonists, receptors and solvent molecules. Disruption of this delicate and fine-tuned equilibrium has potential effects on the agonist-receptor binding affinities and we demonstrated that three outcomes are possible: an increase in the binding affinity for histamine, a decrease for 2-methylhistamine, and no significant effect for 4-methylhistamine. The computational results presented here reveal that the predominance of the deuteration effect is exerted within the receptor binding site, with only a small contribution from the hydration in aqueous solution. Changed geometric parameters and the therewith-associated altered energy of the hydrogen bond upon deuteration is a nuclear quantum effect and has no classical analogue. Therefore, the nature of ligand binding to the receptor has a small but significant nuclear quantum mechanical character. Selective replacement of non-exchangeable hydrogen atoms with deuterium does not significantly impact the pharmacologic profile of drugs and can elongate the duration of action due to slower decomposition. Clinical trials are of deuterated drugs are in progress [62]. There is still a long way toward proper understanding of agonist-induced receptor activation. We are sure that, beside traditional methods of molecular pharmacology, computational work will play an important role in clarifying this process. Multiscale treatment of the receptor activation process is a method of choice. The methodology has to certain extent been developed, is ready to be used [63–66] and will lead towards greater understanding of receptor activation and the design of new drugs.

## Supporting Information

S1 Table PDB structure of the histamine H2 receptor homology model with histamine bound at the binding site. (PDB)
